# Functional intercomparison of intraoperative radiotherapy equipment – Photon Radiosurgery System

**DOI:** 10.1186/1748-717X-2-11

**Published:** 2007-02-27

**Authors:** Kris S Armoogum, John M Parry, Salam K Souliman, David G Sutton, Colin D Mackay

**Affiliations:** 1 Department of Medical Physics, Ninewells Hospital and Medical School, Tayside University Hospitals NHS Trust, Dundee, UK

## Abstract

**Background:**

Intraoperative Radiotherapy (IORT) is a method by which a critical radiation dose is delivered to the tumour bed immediately after surgical excision. It is being investigated whether a single high dose of radiation will impart the same clinical benefit as a standard course of external beam therapy. Our centre has four Photon Radiosurgery Systems (PRS) currently used to irradiate breast and neurological sites.

**Materials and methods:**

The PRS comprises an x-ray generator, control console, quality assurance tools and a mobile gantry. We investigated the dosimetric characteristics of each source and its performance stability over a period of time. We investigated half value layer, output diminution factor, internal radiation monitor (IRM) reproducibility and depth-doses in water. The half value layer was determined in air by the broad beam method, using high purity aluminium attenuators. To quantify beam hardening at clinical depths, solid water attenuators of 5 and 10 mm were placed between the x-ray probe and attenuators. The ion chamber current was monitored over 30 minutes to deduce an output diminution factor. IRM reproducibility was investigated under various exposures. Depth-dose curves in water were obtained at distances up to 35 mm from the probe.

**Results:**

The mean energies for the beam attenuated by 5 and 10 mm of solid water were derived from ICRU Report 17 and found to be 18 and 24 keV. The average output level over a period of 30 minutes was found to be 99.12%. The average difference between the preset IRM limit and the total IRM count was less than 0.5%. For three x-ray sources, the average difference between the calculated and actual treatment times was found to be 0.62% (n = 30). The beam attenuation in water varied by approximately 1/r^3^.

**Conclusion:**

The x-ray sources are stable over time. Most measurements were found to lie within the manufacturer's tolerances and an intercomparison of these checks suggests that the four x-ray sources have similar performance characteristics.

## Background

The past 20 years have seen a distinct shift in the paradigm used in the treatment of breast cancer, away from radical interventions toward more conservative techniques. Randomised clinical trials have shown that breast conserving surgery allied to external beam radiotherapy compares favourably with more radical procedures such as mastectomy [1-9]. However, the external beam radiation fields still encompass all of the breast tissue – healthy and cancerous. Intraoperative radiotherapy, using an innovative miniature x-ray source avoids unnecessary treatment to the whole breast and delivers a critical dose to the tumour bed only. It has also been shown to be effective in the treatment of intracranial malignancies [10-12]. Ninewells Hospital currently uses the Photon Radiosurgery System (PRS – Carl Zeiss Surgical GmbH, Oberkochen, Germany) to treat breast and neurological tumours. The operation of four PRS x-ray sources was compared over a period of six months. The parameters measured were output trends, half value layer (HVL), output diminution factor, internal radiation monitor (IRM) values and depth doses in water.

## Methods

### Device description

The Photon Radiosurgery System includes an x-ray generator capable of delivering a prescribed therapeutic radiation dose directly to the tumour bed during the surgical procedure. The device itself weighs 1.62 kg, has dimensions 17.5 × 11 × 7 cm with a 3.2 mm diameter and 100 mm length chromium nitride coated probe. The X-ray source (XRS) is powered by a portable, electronic control console. The PRS is supplied with a set of components, which facilitate accurate alignment of the XRS probe as well as quality assurance checks [13].

**Figure 1:**
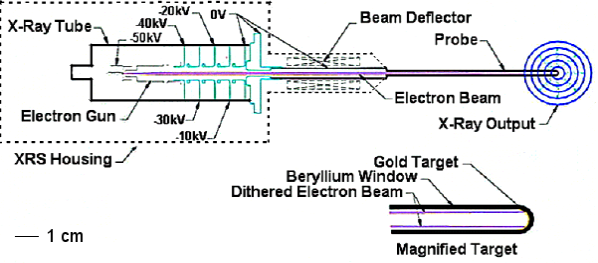
**X-Ray Source (XRS)**. Cross sectional representation of the miniature x-ray source showing the x-ray tube, electron gun, beam deflector and probe. The radiation dose distribution is spherical due to dithering of the electron beam onto the gold target.

As shown in figure 1, an electron beam is accelerated through a high-voltage field (range 30–50 kV in 10 kV increments) and then passes through a deflection chamber to control beam position and to ensure the beam passes down the centre of the probe. The beam current is selectable (5, 10, 20 and 40 μA). After travelling down the evacuated, magnetically shielded probe, the electron beam strikes a thin gold target (1 μm) at the probe tip producing x-ray photons whose mean effective energies are typically in the 5–25 keV range. The distal 20 mm of the probe is fabricated from beryllium (0.5 μm), which is transparent to very low energy x-ray photons [14].

The x-rays are emitted from the tip in a spherical symmetrical pattern resulting in a dose rate in tissue of approximately 120 Gy per hour at 10 mm from the probe tip [14]. Other research has shown that the relative biological effectiveness at clinically relevant doses and dose rates for this very low-energy x-ray source is considerably greater than unity [15-18].

### Output trends

Weekly output constancy checks, based on the manufacturer's recommendations, were carried out on four x-ray sources over an extended period. Very low kV x-rays produced at the tip of the probe are detected by an internal radiation monitor (IRM). The IRM uses a radiation detector that is internal to the XRS and it detects radiation, which passes back along the path of the electron beam. An external radiation monitor (ERM) provides an independent check on the correct performance of the XRS during radiation delivery. It serves as a complementary method of monitoring dose delivery in that it detects scattered radiation exiting the patient. Radiation detected by the ERM is converted to an output pulse rate related to the radiation intensity. At the start of treatment, the output pulse rate from the ERM is measured and provides a baseline value against which successive measurements are compared. The manufacturer's recommended IRM/ERM test procedure was used to check the response of both the internal and external radiation monitors. The ERM was connected to the ERM Test Adapter and docked with the XRS Probe under test.

The test first measures the background counts detected via the IRM and ERM radiation monitors over a 30 second period. The complete test takes approximately 8 minutes and cycles through all the kV and μA combinations including 50 kV and 40 μA. The IRM count rate obtained during this verification procedure is used in treatment administration. The IRM count rate in Hz is multiplied by the treatment time in seconds to give the absolute number of IRM counts at which the treatment is terminated. The output of three x-ray sources was monitored in terms of the internal count rate over a period of 25 weeks. The first value obtained at commissioning was used as the baseline for each x-ray source and subsequent readings were compared to this value.

### Half Value Layer

The HVLs for all the x-ray sources was determined in air by a broad beam method, in which the x-ray probe is placed 20 cm away from the ionisation chamber, with high purity aluminium attenuators placed near the midpoint. The attenuators ranged in thickness from 0.03 to 1.22 mm. We investigated a single x-ray probe to ascertain the effects of beam hardening at clinical depths relevant to our treatment of invasive intracranial malignancies and early stage breast cancer. Solid water WT1 (GAMMEX RMI, Wisconsin, USA) attenuators 5 mm and 10 mm thick were placed between the x-ray probe and the aluminium attenuators, approximately 2 cm away from the probe. The equivalent energies for the unattenuated beam and for the beam attenuated by 5 mm and 10 mm of solid water were derived from tabulated data in ICRU Report 17 [19]

### Output diminution factor

To determine the constancy of output from the x-ray sources, the ion chamber current was monitored over a period of 30 minutes equivalent to typical clinical treatment duration. After the full verification procedure, a parallel plate ion chamber (Type N23342, PTW, Freiburg, Germany) calibrated in terms of exposure was coupled to the PAICH (Probe Adjuster Ion Chamber Holder) and the x-ray source to be tested. The dosemeter (Unidos 10005–50234) was switched on and left for 30 minutes to attain thermal and electronic equilibrium. The settings used were 50 kV and 40 μA to mimic clinical use and the run time set to 32.25 minutes. The uncorrected current readings in pA were taken at 2-minute intervals up to 32 minutes giving 16 readings in all.

### IRM reproducibility and linearity

Reproducibility of the IRM dosimetry system was investigated for the four X-ray sources under various exposures [20]. Exposure was controlled using a pre-set number of IRM counts at a count rate equal to that obtained during the verification of each of the x-ray sources. A beam voltage of 50 kV and a beam current of 40 μA were used. Measurements were made for exposures equivalent to 1, 5, 10 and 15 minutes.

The linearity of the IRM dosimetry system was ascertained at the clinical treatment voltage for the range of beam current options offered by the PRS system. A 50 kV accelerating potential was selected, the number of counts set to 5 × 10^6 ^and beam current chosen as 10, 20 and then 40 μA. For each combination of voltage and current, a full verification of the x-ray source must be performed.

### Reproducibility with probe orientation

A succession of exposures was made with beam parameters of 50 kV and 40 μA. The probe was rotated through 0°, 45°, 90°, 135° and 180° to the vertical plane. Three exposures, each of 2 minutes duration were made with a soft x-ray ionisation chamber placed 20 mm from the probe tip. The mean of three readings in coulombs was calculated at each probe orientation.

### Constancy of treatment delivery time

After a full verification procedure the IRM limit corresponding to 2 minutes was calculated based on the measured IRM rate in hertz. A beam voltage of 50 kV and a beam current of 40 μA were selected and 30 sequential exposures each of 2 minutes duration were made. The actual treatment time, that is the beam-on time was recorded and compared to the preset treatment time.

We have used three x-ray sources clinically for a total of 52 treatments. A hardcopy of the treatment delivery is produced by the PRS controller and includes the actual beam-on time. We compared these with the calculated values entered on the treatment calculation sheets to determine the time divergence under clinical conditions.

### Depth dose in water

**Figure 2:**
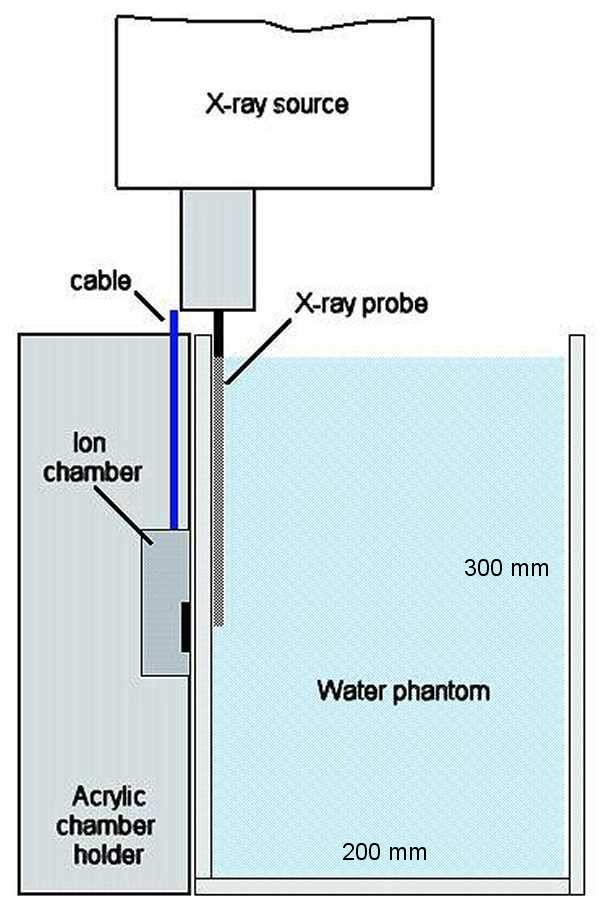
**Set-up used for Depth Dose Measurements**. Experimental set-up used to obtain Depth-Dose curves showing the x-ray source, custom-built water phantom and acrylic backscatter material of dimensions 30 cm × 30 cm × 10 cm. A low kV x-ray parallel plate ionisation chamber (PTW N23342) was used to measure the dose at distances of 10 to 35 mm away from the tip of the probe, in increments of 1 mm.

Depth-dose curves were obtained by measuring the chamber output at distances of between 12 and 35 mm away from the probe tip in a water phantom of dimensions 300 × 300 × 200 mm. The ionisation chamber was placed as close as possible to the outer surface of the water phantom to minimise the effect of air gaps. The position of the x-ray source was then adjusted such that the tip of the probe was just in contact with the inner wall of the phantom and care was taken not to accidentally bend the probe in doing so. The minimum distance away from the probe tip at which the ionisation chamber could be positioned was 12 mm, accounted for by the perspex wall thickness (10 mm) of the custom-built water phantom and the recessed depth (2 mm) of the chamber window as shown in figure 2.

We acquired depth dose data in water to reduce the uncertainties involved in converting from an air measurement to a measurement in water. In addition, a comparison was made between measured depth doses and corresponding values obtained from an analytic fit of each of the depth dose curves [14].

## Results

### Output trends

**Figure 3:**
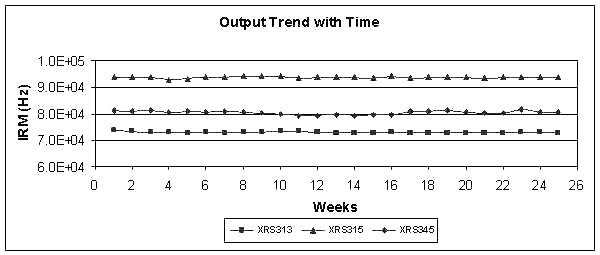
**Output trend with time**. The output of three x-ray sources was monitored over a period of 25 weeks and compared to the baselines values. Although the absolute output of each source is different, the variation from the baseline was less than 1% in each case.

Full system verification was performed on each of three x-ray sources on a weekly basis for 25 weeks and the value of the internal rate monitor after 3 minutes (50 kV, 40 μA) was recorded. Figure 3 shows a plot of the internal count rate against time showed an average variation from the baseline values of 0.67% (range 0.24% – 1.22%).

### Half value layer

**Figure 4:**
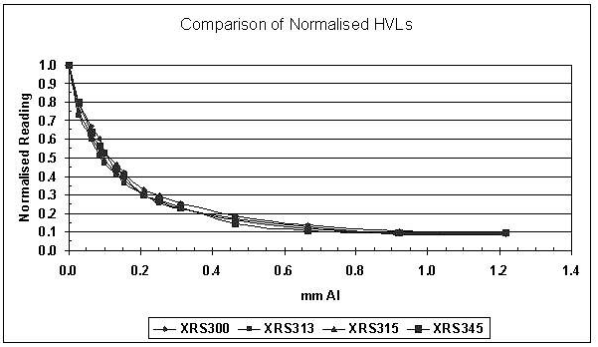
**Comparison of normalised Half Value Layers (HVL)**. The HVLs for all the x-ray sources was determined by a broad beam method, in which the x-ray probe is placed 20 cm away from the ionisation chamber, with high purity aluminium attenuators placed near the midpoint. The HVL for each x-ray source was approximately 0.1 mm Al.

The mean HVL for the four sources was found to be 0.11 mmAl (range 0.10 – 0.12 mmAl) as shown in figure 4. Based on a density of Aluminium of 2.699 × 10^3 ^kg.m^-3 ^and using interpolated mass attenuation coefficients (μ/ρ) from ICRU Report 17, the mean equivalent energy for the four sources was 10.75 keV (range 10.5 – 11.0 keV). This is comparable to the equivalent energy found for a single x-ray source by Beatty *et al *[14].

Measurements made at 5 mm deep in solid water resulted in a first HVL of 0.54 mm Al and an equivalent photon energy of 18.0 keV. At 10 mm deep, beam hardening increased the first HVL to 1.11 mm Al with an equivalent photon energy of 23.5 keV.

### Output diminution factor

**Figure 5:**
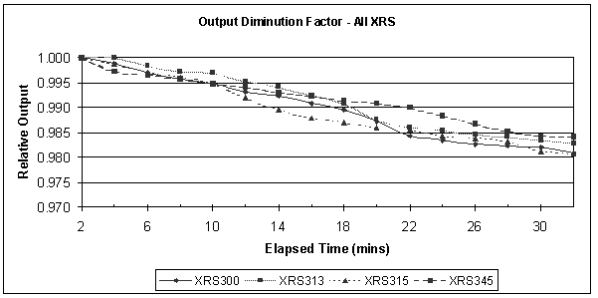
**Output Diminution Factor**. To determine the constancy of output from the x-ray sources, the ion chamber current was monitored over a period of 30 minutes equivalent to typical clinical treatment duration. A difference between the initial and final current readings was consistently observed and the mean reduction was 1.58%.

A plot of output versus elapsed time shows definite, reproducible output reduction as seen in figure 5. A difference between the initial and final current readings was consistently observed and the mean reduction was 1.58%, (range 0.54% – 2.22%). The average output level for all sources over a period of 30 minutes was found to be 99.12%, (range 97.78% – 100%). This reduction in output is small but for the purposes of dose accuracy, should be included in the dose calculation [14].

### IRM reproducibility and linearity

A measure of the internal radiation monitor reproducibility was obtained from the difference between the actual beam-on time and the preset times of 1, 5, 10 and 15 minutes for each x-ray source. The mean difference for the four sources was found to be 0.23% (range 0.13% – 0.35%). For all four sources, a plot of IRM rate versus beam current was found to be linear.

### Reproducibility with probe orientation

For all four x-ray sources, the average difference between the mean reading at each orientation and the overall mean was found to be 0.49% (range 0.42% – 0.64%). This difference is greater than that found in a previous study where a single x-ray source was investigated [14]. The difference can be accounted for by the fact that our measurements were performed at five angles rather than three and we intercompared four x-ray sources.

### Constancy of treatment delivery time

Based on 30 sequential exposures of 2 minutes each, the average difference or all four x-ray sources between the preset and actual treatment times was found to be 0.55% (range 0.41% – 0.66%).

Analysis of the calculated and actual clinical treatment times showed a slightly higher average difference of 0.62% (range 0.53 – 0.74%) over a mean treatment time of 26.27 minutes. This difference arises because x-ray production does not stop instantaneously when the x-ray source is turned off. This in turn is due to short delays, typically less than 1 millisecond, in the electronics that turn the x-rays off. In our clinical experience, the maximum difference between the actual treatment time and the calculated treatment time was 2.65%.

### Depth dose in water

**Figure 6:**
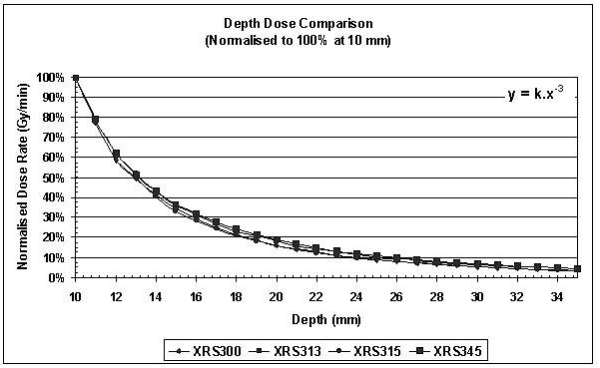
**Depth Dose Comparison**. Depth-dose curves were obtained by measuring the chamber output at distances of between 12 and 35 mm away from the probe tip in water. The ionisation chamber was placed as close as possible to the outer surface of the water phantom to minimise the effect of air gaps. It was found that beam attenuation varies by approximately 1/r^3^as previously reported [14, 20].

Figure 6 shows a comparison of the depth dose curves obtained in water at 50 kV and 40 μA for all four x-ray sources. The dose rate was normalised to 100% at 10 mm deep to mimic the prescription depth we use for breast treatments. It was found that beam attenuation varies by approximately 1/r^3 ^as previously reported [14].

**Figure 7:**
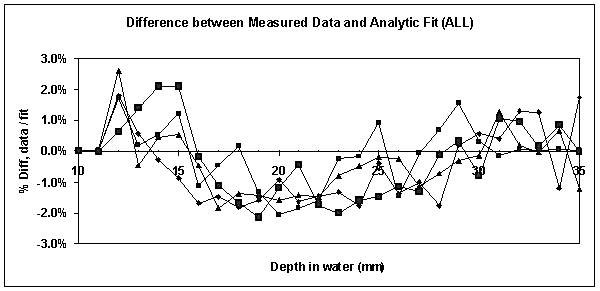
**Difference between measured data and analytic fit**. This figure shows that the percentage difference between the measured depth doses and an analytical (power) fit to the data varied approximately in the range ± 2%, again comparable to the findings by Beatty *et al *[14].

Figure 7 shows that the percentage difference between the measured depth doses and an analytical (power) fit to the data varied in the range ± 2%, again comparable to the findings by Beatty *et al *[14].

## Discussion

This paper describes how we performed a functional intercomparison of four PRS very low kilovoltage intraoperative radiotherapy units used for the treatment of early stage breast cancer and intracranial metastases. Our centre was in a unique position to perform this study due to the fact that we have four of the miniature x-ray sources available. In comparison, most other centres participating in the Targit clinical trial have one or two units.

We are currently treating patients with early stage breast cancer and those with invasive intracranial malignancies. However, we also envisage treating patients with colorectal cancer and cancers of the upper gastrointestinal tract. It is therefore likely that we will need to use different x-ray sources for different treatment sites. This highlights the need to perform a functional intercomparison amongst our four x-ray sources to ensure that the prescribed dose in each case was being delivered, regardless of which x-ray source was used.

We perform a weekly verification procedure based on the manufacturer's recommendations on each x-ray source, and data form this has enabled us to observe the output trends of our sources. Over a six-month period, we observed a deviation from baseline values of less than ± 1% for all sources. This compares favourably with the output constancy of ± 2% recommended in 1997 by the European Commission [21].

In general, due to the rapid dose fall-off from the PRS x-ray sources, absolute dosimetric measurements are difficult to reproduce as the dose in water can vary considerably across the volume of a detector. Further, the low-energy x-ray spectrum hardens rapidly with distance in water and this can affect dosimetric parameters such as energy absorption coefficients and tissue equivalence of phantom materials [22].

In terms of the uncertainty of the depth dose measurements, we quantified it as follows. Temperature and pressure correction factor (± 0.3%), calibration factor of the ion chamber (± 2.2%), calibration factor of the electrometer (± 0.5%), ion chamber current (± 3.4%), output decay factor (± 1.6%), absolute calibration in water (± 9%) and relative dose calibration in water (± 4%). Combining these errors in quadrature results in an overall error of ± 10.8%. This corresponds to a positional uncertainty in the prescribed dose of ± 0.22 mm at a depth of 5 mm increasing to ± 0.44 mm at a depth of 10 mm in water. This shows that the most significant error in determining the output dose lies in the measurement of the position of the chamber relative to the x-ray source.

## Conclusion

We have performed an intercomparison of four miniature, low kV x-ray sources currently used in an international clinical trial for the treatment of early stage breast cancer. The characteristics intercompared were output stability, half value layer, output diminution factor, internal radiation monitor reproducibility and variation of depth doses in water. The x-ray sources have proven to be stable over time. Most measurements were found to lie within the manufacturer's tolerances and an intercomparison of these checks show that the four x-ray sources have similar performance characteristics.

## Competing interests

The author(s) declare that they have no competing interests.

## Authors' contributions

KA conceived of the study, performed the collection, analysis, interpretation of the data and drafted the manuscript. JP critically reviewed the scientific content of this manuscript. CM critically reviewed the scientific content of this manuscript. SS critically reviewed the scientific content of this manuscript. DS critically reviewed the content of this manuscript.
